# Nonselective Beta Blockers Are Beneficial in Patients with Cirrhotic Ascites and Spontaneous Bacterial Peritonitis: A Propensity-Matched Study

**DOI:** 10.3390/jcm15041516

**Published:** 2026-02-14

**Authors:** Ahmad Nawaz, Azhar Hussain, Abdelkader Chaar, Vishnu Charan Suresh Kumar, Ganesh Aswath, Kelita Singh, Hafiz Muzaffar A. Khan, Savio John

**Affiliations:** 1 Division of Gastroenterology, SUNY Upstate Medical University, Syracuse, NY 13210, USA; nawaza@upstate.edu (A.N.); chaara@upstate.edu (A.C.);; 2 Division of Medicine, SUNY Upstate Medical University, Syracuse, NY 13210, USA; 3 Division of Gastroenterology, Staten Island University Hospital, Staten Island, NY 13210, USA

**Keywords:** nonselective beta blockers, liver cirrhosis, spontaneous bacterial peritonitis

## Abstract

**Background:** The role of nonselective beta blockers (NSBB) in patients with cirrhosis and spontaneous bacterial peritonitis (SBP) has been a subject of debate. Conflicting studies exist regarding their impact on mortality in this population. This study aims to evaluate the effect of NSBB on mortality in patients with cirrhotic ascites and a history of SBP. **Methods:** Data were obtained from the TRNETX database, identifying patients aged 18 to 80 years with cirrhosis, ascites, and SBP using ICD-9 and ICD-10 codes. The study period spanned from September 2001 to January 2024. Patients were divided into two groups: those with SBP receiving NSBB (SBP + NSBB), such as carvedilol, nadolol, and propranolol, and those with SBP not receiving NSBB (SBP − NSBB). The primary outcome was all-cause mortality, and the secondary outcome was the development of acute kidney injury (AKI). Outcomes were assessed over a two-year follow-up period. Additionally, we evaluated the association of NSBB use and mortality in cirrhotic ascites by creating two separate cohorts: patients with cirrhotic ascites on NSBB (Ascites + NSBB) and those not on NSBB (Ascites − NSBB). A 1:1 propensity score matching was conducted based on baseline demographics, comorbidities, and laboratory parameters, including creatinine, INR, sodium, albumin, and bilirubin. **Results**: Before propensity matching, 18,160 patients were identified in the SBP-NSBB cohort, and 14,198 patients were in the SBP + NSBB cohort. After matching, each group comprised 11,801 patients. Patients with SBP who did not receive NSBB therapy exhibited higher mortality than those on NSBB therapy [OR 1.12, 95% CI 1.05–1.21]. Conversely, the incidence of AKI was higher in the SBP + NSBB group [OR 0.91, 95% CI 0.87–0.95]. In the cirrhotic ascites cohort, patients not receiving NSBB (Ascites − NSBB) demonstrated higher mortality compared to those on NSBB (Ascites + NSBB) [OR 1.17, 95% CI 1.13–1.20]. **Conclusions**: In a propensity-matched analysis of large patient cohorts, NSBB therapy was associated with reduced mortality in both patients with cirrhotic ascites and those with SBP. Despite a higher incidence of AKI in the SBP + NSBB group, NSBB treatment appears beneficial in reducing overall mortality in these populations.

## 1. Introduction

Nonselective beta blockers (NSBB) have conventionally been used in patients with cirrhotic ascites and decompensated liver cirrhosis for primary or secondary prophylaxis of variceal hemorrhage, as long as the baseline heart rate, blood pressure, and renal function of the patients are within acceptable limits [1,2]. NSBB may be beneficial in decompensated cirrhosis with ascites and a history of spontaneous bacterial peritonitis (SBP) due to two major effects: reduction in the severity of portal hypertension and a potential decrease in chronic inflammation—both of which are key determinants of decompensation and mortality in liver cirrhosis [3,4]. Liver cirrhosis induces a chronic inflammatory state driven by the release of cytokines and activation of beta-2 receptors, which can be blocked by NSBB. Additionally, NSBB may positively impact this chronic inflammatory syndrome by improving intestinal barrier function, reducing bacterial translocation, and modulating immune system activation [5]. The clinical application of the nonselective beta blockers (NSBBs) in decompensated liver cirrhosis has evolved significantly from the “window hypothesis”, which initially suggested that NSBBs might increase the mortality in patients with refractory ascites or spontaneous bacterial peritonitis. However, recent American Association for the Study of Liver Diseases (AASLD) guidelines have shifted this paradigm, endorsing more personalized approach [6,7,8]. Current AASLD guidelines suggests that NSBBs should be considered even in advanced stages to prevent further decompensation and improve survival, provided that systemic hemodynamics are persevered.

There is conflicting data regarding the use of NSBB in patients with cirrhosis and SBP, particularly concerning mortality and renal adverse events. A 2014 study reported an increased incidence of acute kidney injury (AKI), hepatorenal syndrome (HRS), and higher mortality in cirrhotic patients with ascites on NSBB [9]. Conversely, a 2016 study demonstrated reduced mortality in patients on propranolol following SBP [2]. Further, a 2019 study showed that NSBB was associated with improved 28-day transplant-free survival, with renal impairment observed only in SBP patients with a mean arterial pressure (MAP) below 65 mmHg [10]. Furthermore, the ATTIRE clinical trial highlighted the risks of aggressive hemodynamic interventions. This trial underscores the fragility of the circulatory system in patients with decompensated liver cirrhosis and SBP, and reinforces the importance of the cautious use and titration of the various medications, including NSBBs [11].

Given the scarcity and inconsistency of data regarding the use of NSBBs in cirrhotic patients with a history of SBP, this study aims to investigate the impact of NSBBs on mortality in patients with cirrhotic ascites and a history of SBP.

## 2. Materials and Methods

### 2.1. Study Population and Design

This is a retrospective, propensity-matched cohort study utilizing data from the US Collaborative Network within the TriNetX Research Network (Cambridge, MA, USA). We identified patients aged 18 to 80 years with liver cirrhosis between September 2001 and January 2024. The laboratory values, baseline demographics and comorbidities were captured at the time of NSBB initiation. All data were de-identified and anonymized, with no patient, institutional, or geographical identifiers. The dataset includes contributions from 61 healthcare organizations, encompassing large healthcare centers, community setups, and subspecialty clinics. Data were retrieved from Electronic Health Records (EHRs) and insurance records, covering both inpatient and outpatient settings, to reflect real-world clinical scenarios.

A detailed description of the data source, study definitions, inclusion and exclusion criteria, and relevant codes (ICD and CPT) used to query the TriNetX Research Network database is provided in the (Table 1).

### 2.2. Study Cohort Selection

#### 2.2.1. Inclusion Criteria

- Patients aged 18 to 80 years with liver cirrhosis of any etiology (Table 1).

#### 2.2.2. Treatment Groups

- **Cirrhosis + Ascites + SBP − NSBB**: Patients with liver cirrhosis, ascites, and SBP who were not on NSBB (carvedilol, nadolol, or propranolol).
- **Cirrhosis + Ascites + SBP + NSBB**: Patients with liver cirrhosis, ascites, and SBP who received NSBB therapy (carvedilol, nadolol, or propranolol).

All patients were followed until death or for a minimum of two years.

### 2.3. Primary Outcome

- All-cause mortality

### 2.4. Secondary Outcome

Acute kidney injury (AKI) occurred anytime during the study period.

Clinical outcomes were measured after 1:1 propensity score matching based on baseline demographics and comorbidities (e.g., decompensated heart failure, coronary artery disease, ischemic or non-ischemic cardiomyopathy, constrictive cardiomyopathy, restrictive cardiomyopathy, dilated cardiomyopathy, hypertension, hypotension, cardiogenic syncope, history of any malignancy), and laboratory values (creatinine, INR, sodium, albumin, and bilirubin) to balance the severity of liver disease.

### 2.5. Subgroup Analysis

We further analyzed primary and secondary outcomes in patients with cirrhotic ascites by creating two additional groups:

- **Cirrhosis + Ascites − NSBB**: Patients with cirrhosis and ascites not receiving NSBB.
- **Cirrhosis + Ascites + NSBB**: Patients with cirrhosis and ascites on NSBB.

Outcomes (mortality and AKI) were measured after 1:1 propensity matching based on baseline demographics, comorbidities, and laboratory values (creatinine, INR, sodium, albumin, and bilirubin) to balance the severity of liver disease across groups.

### 2.6. Statistical Analysis

To minimize selection bias and control for confounders, we used the TriNetX built-in propensity score matching algorithm. A 1:1 ratio was used to match cohorts by age, gender, race, comorbidities and lab values (creatinine, INR, sodium, albumin, and bilirubin). Standardized differences (Std diff.) were used to assess the effectiveness of matching, with a value below 0.1 indicating minimal and insignificant differences between the cohorts, demonstrating successful matching.

Baseline demographics and clinical characteristics of the propensity-matched cohorts were compared using the Chi-square or Fisher’s exact test for categorical variables, and independent sample t-tests for continuous variables. Odds ratios (OR) and hazard ratios (HR) with 95% confidence intervals (CI) were calculated to compare outcomes between the groups.

## 3. Results

### 3.1. Demographics and Baseline Characteristics

A total of 18,160 patients were identified in the SBP − NSBB cohort, and 14,198 patients in the SBP + NSBB cohort before propensity score matching. After matching, each group comprised 11,801 patients. Baseline characteristics are summarized in Table 2.

The Model for End-Stage Liver Disease (MELD) score, calculated using mean values of total bilirubin, INR, and creatinine, was 24 in both groups before propensity matching.

In the broader cohort of cirrhotic patients with ascites, 199,914 patients were identified in the Cirrhosis + Ascites − NSBB group and 104,188 in the Cirrhosis + Ascites + NSBB group. After propensity matching, both groups contained 95,315 patients each. The mean MELD score was 19 for the Cirrhosis + Ascites − NSBB group and 20 for the Cirrhosis + Ascites + NSBB group before matching. Baseline characteristics for these cohorts are presented in Table 3.

### 3.2. Outcomes

**Figure 1:**
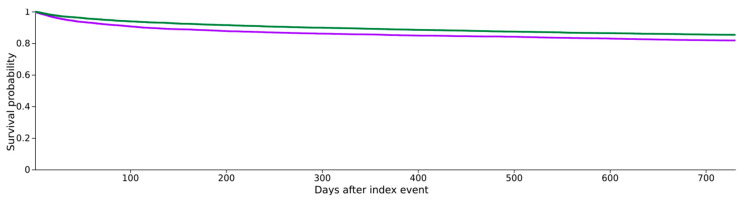
Kaplan–Meier survival analysis for patients with cirrhosis and SBP on NSBB and cirrhotic patients with SBP not on NSBB. Cirrhosis + Ascites + SBP − NSBB—purple; Cirrhosis + Ascites + SBP + NSBB—green.

Higher mortality was observed in the SBP − NSBB group compared to the SBP + NSBB group [OR 1.12, 95% CI 1.05–1.21] (Figure 1).

However, the incidence of AKI was higher in the SBP + NSBB group [OR 0.91, 95% CI 0.87–0.95] compared to the SBP − NSBB group (Table 4).

**Figure 2:**
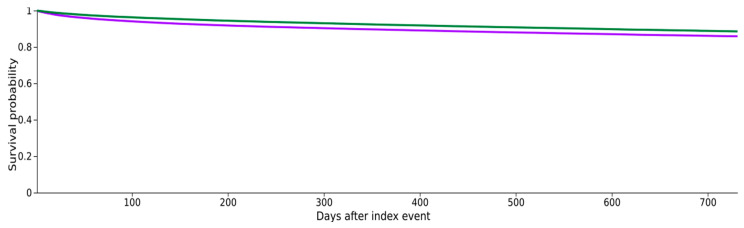
Kaplan–Meier survival analysis for patients with cirrhosis on NSBB and cirrhotic patients who are not on NSBB. Cirrhosis + Ascites − NSBB—purple; Cirrhosis + Ascites + NSBB—green.

In the cohort of patients with cirrhosis and ascites, higher mortality was observed in the Ascites − NSBB group compared to the Ascites + NSBB group [OR 1.17, 95% CI 1.13–1.20] (Table 4, Figure 2).

## 4. Discussion

The rising prevalence of liver cirrhosis and associated complications such as portal hypertension, ascites, and SBP pose increasing challenges in clinical management. This study aimed to evaluate the impact of nonselective beta blockers (NSBB) on mortality in patients with cirrhotic ascites and a history of SBP [12,13,14]. Using real-world data, we demonstrated a significant reduction in mortality among cirrhotic patients with SBP who were treated with NSBB compared to those who were not. These findings are based on large, propensity-matched cohorts comprising 11,801 patients in each group.

Previous studies have presented conflicting data on the use of NSBB in cirrhotic ascites, particularly regarding mortality outcomes [1,2,3]. Our study aligns with findings that indicate lower mortality in patients treated with NSBB. Specifically, our results show that patients with cirrhotic ascites and a history of SBP who were not on NSBB had a higher mortality risk [OR 1.14, 95% CI 1.05–1.24].

Conflicting reports also exist regarding the incidence of acute kidney injury (AKI) in cirrhotic patients on NSBB [12]. Some studies have suggested a three- to five-fold increase in the risk of hepatorenal syndrome (HRS) and AKI in patients on NSBB [12,13,14], while others report a lower incidence of AKI requiring hospitalization in patients with cirrhotic ascites treated with NSBB [15]. In our study, the incidence of AKI was indeed higher in the SBP + NSBB group compared to the SBP − NSBB group. This finding may be explained by the severity of liver disease and hepatic and systemic decompensation in patients with SBP in the context of an acute infection. NSBB use in cirrhotic patients without ascites has been associated with lower odds of AKI; however, cirrhotic patients with ascites and a history of SBP are at a much higher risk of developing AKI. This increased risk may result from complications associated with severe infections like SBP in patients with decompensated liver disease, manifesting as systemic illness. Additionally, the heightened risk in these patients may stem from increased splanchnic vasodilation, sympathetic nervous system activation, and renin–angiotensin–aldosterone system (RAAS) activation unique to this patient population [14,15]. Similarly, recent AASLD guidelines also recommend cautious approach to the use and dose titration of the NSBBs in liver cirrhosis as they may increase the risk of hepatorenal syndrome (HRS-AKI) and mortality [6,7,8]. In fact, guidelines endorse that NSBBs dose should be reduced or be held if patient develops persistent hypotension, progressive hyponatremia, or development of hepatorenal syndrome (HRS-AKI). Our findings support this paradigm from real-world data, demonstrating survival benefits of NSBBs even in patients with history of SBP, provided that clinicians remain vigilant for the adverse events, especially renal adverse events.

Our study has several strengths. Leveraging a patient database based on ICD-coded diagnoses of decompensated cirrhosis provides significant advantages, especially when large-scale randomized controlled trials (RCTs) are impractical due to the high mortality and morbidity associated with the disease. A key benefit is the ability to analyze large, real-world patient populations, enabling more generalizable conclusions. This approach facilitates the study of rare or severe outcomes that might be missed in smaller RCTs and allows for the examination of long-term trends through longitudinal data collection, offering insights into disease progression and treatment outcomes over time. Furthermore, database studies provide an ethical and practical alternative to RCTs, which can be difficult to conduct for critically ill patients. The use of existing clinical data reduces the need for direct patient involvement, avoiding the complexities of randomization while still capturing a broad spectrum of disease severity and comorbidities. This observational method mirrors real-world treatment decisions, making the results applicable to a wider patient population and enhancing understanding of therapeutic effectiveness across diverse clinical settings.

Our study has several limitations. We lacked data on the Child–Turcotte–Pugh (CTP) classification of patients and could not assess the precise duration of NSBB therapy. However, we performed propensity matching using key variables such as total bilirubin, INR, sodium, creatinine, and albumin to mitigate this limitation by accounting for all variables in both the original MELD and MELD 3.0 scores. Although the representative MELD scores (calculated using mean values of total bilirubin, INR, and serum creatinine) were similar between the SBP + NSBB and SBP − NSBB groups, the Cirrhosis + Ascites + NSBB group had a higher MELD score than the Cirrhosis + Ascites − NSBB group. To address the limitation regarding therapy duration, we included only patients with a follow-up period of at least two years or death, ensuring adequate observation time after the initiation of therapy.

Additionally, our dataset lacked information on concomitant treatments for decompensated cirrhosis, including diuretic use, large-volume paracentesis, albumin supplementation, and alcohol consumption history, as these factors could not be captured using ICD codes. Also, due to the database nature of the study, we acknowledge that this study does not provide definitive insights into the specific subgroups like those with refractory ascites or compromised cardiac reserve. Although we carefully matched for MELD score and baseline laboratory and clinical disease characteristics and comorbidities, it has inherent limitations due to its retrospective nature and lacks granular data about amount of alcohol consumption, etiology of liver cirrhosis, particular NSBB’s dose and its dose titration based on their response to treatment and adverse effects experienced during the study period. Aslo, reliance on ICD codes may introduce the selection bias or misclassification bias. These missing variables could introduce confounders or influence outcomes related to NSBB use and associated adverse events, including AKI. Consequently, these limitations may affect the generalizability of our findings. Prospective studies and clinical trials are needed to evaluate the optimal NSBB selection, and dose titration depending on the patients’ disease characteristics and clinical comorbidities, e.g., heart failure.

## 5. Conclusions

Our large, propensity-matched cohort study demonstrates that NSBB use in patients with cirrhotic ascites and a history of SBP is associated with lower all-cause mortality, but a higher incidence of AKI. Future well-controlled, prospective studies are necessary to further delineate the role of NSBB in the management of decompensated cirrhosis with ascites and SBP.

## Figures and Tables

**Table 1:** Complete list of relevant ICD and CPT codes used to query the TriNetX Research Network database.

	ICD 10/ICD 9/Procedure Codes
Liver Cirrhosis	ICD10 K71.7 K74.3 K74.4 K74.5 K74.6 K76.1 K70.11 K70.30 K70.31 K74.00 ICD 9 571 571.5
SBP	ICD 10 K65.2 ICD 9 567.23
Ascites	ICD 10 K70.31 K70.11 R18 R18.8 ICD 9 789.5
Death	ICD10 R99-R99
AKI	ICD10 N17 N17.0 N17.1 N17.2 N17.8 N17.9 ICD 9 584 584.5 584.6 584.7 584.8

**Table 2:** Baseline characteristics of patients with a history of SBP and liver cirrhosis patients with ascites with and without NSBB, before and after a 1:1 propensity matching of the groups based on baseline demographics, comorbidities, and laboratory values.

Variable	Patient Count Before Matching				Patient Count After Matching			
	Cirrhosis + Ascites + SBP − NSBB n = 18,160	Cirrhosis + Ascites + SBP + NSBB n = 14,198	*p*-Value	Std. Diff.	Cirrhosis + Ascites + SBP − NSBB n = 11,801	Cirrhosis + Ascites + SBP + NSBB n = 11,801	*p*-Value	Std. Diff.
Age, Mean ± SD	54.5 ± 11.7	55.4 ± 10.9	<0.001	0.079	55.6 ± 11.4	55.4 ± 10.9	0.029	0.028
White, n (%)	12,245 (67.4)	9729 (68.5)	0.036	0.023	8132 (68.9)	8104 (68.7)	0.694	0.005
Male, n (%)	11,019 (60.7)	9284 (65.4)	<0.001	0.098	7196 (61.0)	7237 (61.7)	0.584	0.007
Diseases of the circulatory system, n (%)	11,516 (63.4)	12,712 (89.5)	<0.001	0.647	10,327(87.5)	10,335 (87.6)	0.875	0.002
Neoplasms, n (%)	5625 (31.0)	75,683 (40.0)	<0.001	0.190	5625 (39.6)	5683 (39.4)	0.831	0.003
Sodium, Mean ± SD	134.4 ± 5.2	135.0 ± 5.2	<0.001	0.119	134.4 ± 5.2	135.0 ± 5.1	<0.001	0.115
Creatinine, Mean ± SD	2.0 ± 9.1	1.9 ± 6.6	0.237	0.015	1.7 ± 5.7	2.0 ± 7.2	0.002	0.044
Total Bilirubin, Mean ± SD	5.0 ± 7.0	4.2 ± 6.0	<0.001	0.117	4.9 ± 7.0	4.3 ± 6.1	<0.001	0.087
INR, Mean ± SD	1.6 ± 0.8	1.6 ± 0.9	0.004	0.037	1.6 ± 0.9	1.6 ± 0.8	0.002	0.045
Albumin, Mean ± SD	2.9 ± 0.7	2.9 ± 0.7	<0.001	0.047	2.9 ± 0.7	2.9 ± 0.7	0.021	0.033

**Table 3:** Baseline characteristics of patients with a history of liver cirrhosis with and without NSBB, before and after a 1:1 propensity matching of the groups based on baseline demographics, comorbidities, and laboratory values.

Variable	Patient Count Before Matching				Patient Count After Matching			
	Cirrhosis + Ascites − NSBB n = 199,914	Cirrhosis + Ascites + NSBB n = 104,188	*p*-Value	Std Diff.	Cirrhosis + Ascites − NSBB n= 95,315	Cirrhosis + Ascites + NSBB n= 95,315	*p*-Value	Std Diff.
Age, Mean ± SD	55.7 ± 11.9	56.5 ± 11.1	<0.001	0.067	57.0 ± 11.4	56.7 ± 11.1	<0.001	0.031
White, n (%)	128,223 (64.8)	68,289 (65.8)	<0.001	0.019	63,781 (66.9)	62,664 (65.7)	<0.001	0.025
Male, n (%)	114,755 (58.0)	65,812 (63.4)	<0.001	0.110	58,560 (61.4)	57,934 (60.8)	0.003	0.013
Diseases of the circulatory system, n (%)	98,888 (50.0)	83,739 (80.6)	<0.001	0.680	75,142(78.8)	75,204 (78.9)	0.728	0.002
Neoplasms, n (%)	55,597 (31.0)	35,286 (34.0)	<0.001	0.127	35,473 (37.2)	34,882 (36.6)	0.005	0.013
Sodium, Mean ± SD	136.6 ± 4.6	136.6 ± 4.6	0.739	0.002	136.6 ± 4.6	136.6 ± 4.6	0.050	0.010
Creatinine, Mean ± SD	1.6 ± 7.4	1.8 ± 7.0	<0.001	0.026	1.5 ± 5.7	1.9 ± 7.3	<0.001	0.050
Bilirubin total, Mean ± SD	2.8 ± 5.2	2.8 ± 4.6	0.896	0.001	2.8 ± 5.2	2.8 ± 4.5	0.963	<0.001
INR, Mean ± SD	1.4 ± 0.9	1.4 ± 0.7	<0.001	0072	1.4 ± 0.8	1.4 ± 0.7	<0.001	0.067
Albumin, Mean ± SD	3.2 ± 0.8	3.1 ± 0.7	<0.001	0.108	3.2 ± 0.8	3.1 ± 0.7	<0.001	0.069

**Table 4:** Effect of beta blockers on renal adverse events and mortality in patients with liver cirrhosis with ascites, patients with a history of SBP, and on liver cirrhosis patients with ascites after a 1:1 propensity matching of the groups based on baseline demographics, comorbidities, and laboratory values.

Outcomes	Cirrhosis + Ascites + SBP − NSBB	Cirrhosis + Ascites + SBP + NSBB	Risk Difference (95% CI)	Risk Ratio (95% CI)
AKI	46.2%	48.3%	−0.020 (−0.033–0.007), *p* < 0.01	0.95 (0.93–0.97)
Death	11.5%	10.3%	0.011 (0.003–0.019), *p* < 0.01	1.11 (1.03–1.19)
**Outcomes**	**Cirrhosis + Ascites − NSBB**	**Cirrhosis + Ascites + NSBB**	**Risk Difference** **(95% CI)**	**Risk Ratio** **(95% CI)**
AKI	31.5%	35.4%	−0.039 (−0.044–0.035), *p* < 0.001	0.88 (0.87–0.90)
Death	9.6%	8.3%	0.013 (0.010–0.015), *p* < 0.001	1.15 (1.21–1.18)

**Abbreviations:** AKI: acute kidney injury, SBP: spontaneous bacterial peritonitis.

## Data Availability

Data is available publicly at the TriNetX database website.

## Abbreviations

AKI	Acute Kidney Injury
CI	Confidence Intervals
CPT	Current Procedural Terminology
EHRs	Electronic Health Records
HCOs	Health Care Organizations
HR	Hazard Ratios
HRS	Hepatorenal Syndrome
ICD	International Classification of Diseases
MAP	Mean arterial pressure
NSBB	Nonselective beta blockers
OR	Odds ratios
RAAS	Renin-angiotensin-aldosterone system
RCT	Randomized Controlled Trials
SBP	Spontaneous Bacterial Peritonitis
